# Alzheimer's disease traits in Parkinson's disease without α‐synuclein seeding

**DOI:** 10.1002/alz.70284

**Published:** 2025-05-19

**Authors:** Bárbara Fernandes Gomes, Carly M. Farris, Yihua Ma, Luis Concha‐Marambio, Johanna Nilsson, Karin Forsberg, Russ Lebovitz, Ulf Andreasson, Kaj Blennow, Henrik Zetterberg, David Bäckström

**Affiliations:** ^1^ Department of Psychiatry and Neurochemistry Institute of Neuroscience and Physiology the Sahlgrenska Academy at the University of Gothenburg Mölndal Sweden; ^2^ R&D Unit, Amprion Inc. San Diego California USA; ^3^ Department of Clinical Science Neurosciences Umeå University Umeå Sweden; ^4^ Clinical Neurochemistry Laboratory Sahlgrenska University Hospital Mölndal Sweden; ^5^ Paris Brain Institute ICM Pitié‐Salpêtrière Hospital Sorbonne University Paris France; ^6^ Neurodegenerative Disorder Research Center Division of Life Sciences and Medicine and Department of Neurology Institute on Aging and Brain Disorders University of Science and Technology of China and First Affiliated Hospital of USTC Hefei P.R. China; ^7^ Department of Neurology Sahlgrenska University Hospital Gothenburg Sweden; ^8^ Department of Neurodegenerative Disease UCL Institute of Neurology, Queen Square London UK; ^9^ UK Dementia Research Institute at UCL London UK; ^10^ Hong Kong Center for Neurodegenerative Diseases Clear Water Bay Hong Kong China; ^11^ Wisconsin Alzheimer's Disease Research Center University of Wisconsin School of Medicine and Public Health University of Wisconsin–Madison Madison Wisconsin USA

**Keywords:** Alzheimer's disease, fluid biomarker, Parkinson's disease, seed amplification assay, synucleinopathy

## Abstract

**INTRODUCTION:**

The α‐synuclein (αSyn) seed amplification assay (αSyn‐SAA) is an accurate tool to detect αSyn seeds in patients with Parkinson's disease (PD). However, a minority of clinically diagnosed PD patients are negative for αSyn.

**METHODS:**

The αSyn‐SAA was performed in cerebrospinal fluid (CSF) of individuals with PD (*n* = 93), multiple system atrophy (MSA, *n* = 26), progressive supranuclear palsy (PSP, *n* = 18), corticobasal syndrome (*n* = 3), and healthy controls (*n* = 29).

**RESULTS:**

The αSyn‐SAA detected αSyn in 90% of PD and 81% of MSA patients, while exhibiting high specificity (97%). SAA– PD patients had a distinct phenotype compared to SAA+ PD, including a marked postural instability/gait disorder (*P *= 0.002), impaired episodic memory, and lower CSF amyloid beta_42_ (*P *= 0.03). SAA+ PSP also displayed distinctive traits.

**DISCUSSION:**

A negative αSyn‐SAA in PD is associated with a distinct phenotype and pathological findings suggesting that these patients may have a motor subtype of Alzheimer's disease. This could influence future clinical trials.

**Highlights:**

- The α‐synuclein seed amplification assay (αSyn‐SAA) is a robust assay.
- αSyn‐SAA–negative Parkinson's disease shows a distinct motor–cognitive phenotype.
- Autopsy showed Alzheimer's disease (AD) pathology in parkinsonian diseases.
- AD stands as a major clinical confounder for the diagnosis of movement disorders.

## 1 INTRODUCTION

Parkinson's disease (PD) is the second most common neurodegenerative disorder, after Alzheimer's disease (AD), estimated to affect 1% to 2% of individuals > 65,^1^ a number expected to increase as the population ages.

Idiopathic PD is a synucleinopathy and, as such, defined by the presence of α‐synuclein (αSyn) inclusions in neuronal cells and processes, known as Lewy bodies (LBs) and Lewy neurites, respectively.^2^ This pathological αSyn accumulation ultimately leads to the neurodegeneration of the nigrostriatal pathway (another PD hallmark), resulting in typical motor signs and symptoms like bradykinesia, tremor, and rigidity—collectively referred to as parkinsonism.^3^ However, PD is a heterogeneous disease, encompassing a variety of clinical presentations. PD patients can often be categorized into motor disease subtypes—for example, in tremor‐ or postural instability and gait difficulty (PIGD)‐dominant variants, or a mixed phenotype presenting with both symptom types.^4^ While the tremor‐dominant subtype present with resting tremor and an overall more benign disease course, PIGD‐PD often occurs in older patients and has a worse prognosis, with higher incidence of cognitive impairment, dementia, and rapid disease progression.^4^ Also contributing to PD heterogeneity are comorbid neurodegenerative diseases and genetic factors, where monogenetic mutations account for ≈ 5% to 10% of all PD cases.^5^ Advances in the understanding of the biological underpinnings of PD and improved technology for detecting pathological αSyn has led to calls for a biological, rather than solely clinical, definition of the disease.^6^, ^7^

However, while features like parkinsonism and LB pathology are all hallmark indicators of PD, they are not exclusive to this disease. Both parkinsonism and LB are present in other synucleinopathies, such as dementia with Lewy bodies (DLB) and multiple system atrophy (MSA). MSA is also a synucleinopathy, but, unlike PD and DLB, it is characterized by cytoplasmic αSyn inclusions in glia instead of exclusively in neuronal cells.^8^ Additionally, the type of αSyn seed strain observed in MSA differs from that in PD and DLB.^9^, ^10^ Parkinsonism also occurs in progressive supranuclear palsy (PSP) and corticobasal syndrome (CBS) in which the main pathological hallmark is tau and not αSyn.^11^, ^12^ Moreover, studies report LBs in the brain of 10% to 30% of pathologically confirmed PSP patients, suggesting comorbid LB pathology in PSP is more common than previously thought.^13^, ^14^ While DLB, MSA, PSP, and CBS present similarities to PD, they are considered atypical parkinsonian diseases due to their rapid and aggressive disease progression and limited response to levodopa treatment.^15^ Finally, both parkinsonism and αSyn inclusions can be found in AD. Studies report up to 90% of AD patients to display parkinsonian features (usually the akinetic–rigid parkinsonism type) at different disease stages, and that > 50% of patients have concomitant LB pathology.^16^, ^17^, ^18^, ^19^, ^20^, ^21^, ^22^ Molecularly, studies investigating neurodegeneration have pointed toward a synergistic relationship between AD and PD.^22^ However, while extrapyramidal signs occur in AD, they are not generally considered to manifest in a progressive and levodopa‐responsive manner, as would a PD‐like disorder.^21^

The clinical and molecular overlap between these different neurodegenerative diseases poses a considerable diagnostic challenge, particularly in early stages,^23^ contributing to a significant rate of misdiagnosis. This underscores the need for biomarkers that can support the clinical diagnosis in living patients.

The αSyn seed amplification assay (αSyn‐SAA) has been shown to be a robust and highly accurate method to detect misfolded αSyn in cerebrospinal fluid (CSF), with numerous studies pointing to its potential utility in clinical routine, highlighting its high sensitivity and specificity.^24^, ^25^, ^26^, ^27^, ^28^, ^29^, ^30^ Still, in most of the aforementioned studies, a small subset of clinically diagnosed idiopathic PD patients is negative for the presence of αSyn seeds in CSF, based on the αSyn‐SAA. There is an ongoing debate as to whether such patients should be classified as having PD.

Given these unanswered questions, this study aimed at using an optimized αSyn‐SAA to assess detection of αSyn aggregation in CSF of patients with well‐established diagnoses of PD and other parkinsonian diseases, agreement with autopsies, and to characterize patients at baseline and longitudinally according to their synuclein aggregation status.

## 2 METHODS

### 2.1 Participants

Consecutive patients with a well‐established diagnosis of PD (*n* = 93), MSA (*n* = 26), PSP (*n* = 18), or CBS (CBS, *n* = 3), from the New Parkinsonism in Umeå (NYPUM, *n* = 94 and ParkNy, *n* = 46) cohorts—two prospective, academic study cohorts of patients with incident, drug‐naive idiopathic parkinsonism from the Umeå University Hospital catchment region in northern Sweden were included. Twenty‐nine healthy control (HC) participants—volunteers matched by age and sex to the first 50 patients included in the NYPUM study—from the same area as the patients were also included. The NYPUM study is a population‐based study of unselected, idiopathic parkinsonism in the southernmost county of the region. The ParkNy cohort also included—in this study—unselected patients from the same region with MSA or PSP who had been referred to the clinic, which is the region's only neurology clinic as well as the only center for movement disorders. All diagnoses were established by consensus between at least two experienced movement disorder neurologists according to clinical diagnostic criteria at the latest follow‐up. Patients with PD were diagnosed according to the UK Parkinson's Disease Society Brain Bank (UKPDSBB) criteria,^31^ patients with MSA were diagnosed according to consensus criteria for MSA,^32^ patients with PSP were diagnosed according to the National Institute of Neurological Diseases and Stroke and Society for PSP (SPSP) criteria for PSP,^33^ and CBS patients were diagnosed according to Armstrong's criteria.^11^ Requirements for controls were that they had no neurological disorders, had a normal neurological exam, and had normal radiological assessment of presynaptic nigrostriatal integrity using ^123^I‐FP‐CIT SPECT (DATSCAN) imaging. Diagnoses were annually reassessed over the course of follow‐up and, for this study, also validated against the most recent clinical consensus criteria for PD, MSA, and PSP,^32^, ^34^, ^35^ and in several cases by *post mortem* pathology examination (see below). These validated clinical diagnoses were used for assessing αSyn‐SAA performance. All patients underwent a battery of tests to assess motor and cognitive function. Motor performance was examined using the Unified Parkinson's Disease Rating Scale (UPDRS), and in the NYPUM study, its subscores were divided into tremor (sum of Items 20 and 21) and postural instability and gait difficulty (PIGD, sum of Items 13–15, 29, and 30).^36^ Balance and mobility were assessed using the Timed Up and Go (TUG) test, in which the time it takes for a patient to rise up from a chair, walk 3 m, turn around, walk back to the chair, and sit down again is measured.^37^ All patients underwent a neurological exam and modified Hoehn and Yahr (H&Y) scale and oculomotor assessment. In both NYPUM and ParkNy, cognition was assessed using the Mini‐Mental State Examination (MMSE) and all cognitive domains were tested specifically,^38^ of which only episodic memory and language were investigated here. An mild cognitive impairment (MCI) or PD dementia (PDD) diagnosis was made according to Movement Disorder Society (MDS) criteria and based on the overall performance and the cognitive test results.^39^, ^40^ Olfactory function was investigated by the 12‐item Brief Smell Identification Test (BSIT) and smoking habits were recorded.^41^ Patients underwent ancillary investigations, including structural magnetic resonance imaging, sleep analysis, and dysautonomia screening tests, and in atypical cases positron emission tomography (PET). In NYPUM, patients also underwent magnetic resonance diffusion tensor imaging (DTI) and ¹^2^ ^3^I‐IBZM scintigraphy. All investigations and diagnoses were made blinded to αSyn‐SAA results (before they were available). The number of patients per follow‐up time point can be found in Table S1 in supporting information.

**RESEARCH IN CONTEXT**

1. **Systematic review**: The authors used the α‐synuclein seed amplification assay (αSyn‐SAA) to detect the presence of αSyn seeds in patients with Parkinson's disease (PD), multiple system atrophy (MSA), progressive supranuclear palsy (PSP), corticobasal syndrome, and healthy controls.
2. **Interpretation**: The authors found that, based on clinical features and autopsy findings, SAA– PD and SAA+ PSP (i.e., SAA‐incongruent) patients have a high degree of Alzheimer's disease (AD) co‐pathology. In SAA– PD, late‐onset AD with motor symptoms may mimic PD.
3. **Future directions**: Our findings suggest that the αSyn‐SAA is a robust assay that may be used to better diagnose patients with movement disorders. In addition, its use will be beneficial to the correct stratification and eligibility of patients for clinical trials.

### 2.2 Dopamine transporter imaging

Of the patients enrolled in the study, 168 (99%) underwent DATSCAN (GE Healthcare BV) and all but two (one PD patient [Patient 1], discussed below, and one early MSA patient who, however, had a pathological IBZM scintigraphy) were pathological. DAT imaging was done 3 hours after an intravenous bolus dose of 185MBq ^123^I‐FP‐CIT, prior to commencement of medication at baseline. The imaging protocol was done within the framework of a non‐profit clinical trial (EU no. 2009‐011748‐20), as described earlier and constituted a substudy within the research project.^42^ Semiquantitative analysis (based on regions of interest) and visual evaluation of the DAT SPECT were done unbiased by any clinical information. Normal reference values were derived from the age‐matched group of healthy individuals participating in the study, and reduction of DAT uptake in the patients with PD was measured in percent and standard deviation of the normal values. The most affected side (left or right) was defined by the putamen and caudate that showed the largest reduction of ^123^I‐FP‐CIT uptake.

### 2.3 CSF analyses

CSF was collected by lumbar puncture close to the baseline visit and samples were stored at –80°C until analysis. Other fluid biomarkers were analyzed by sandwich enzyme‐linked immunosorbent assay (ELISA) at the University of Gothenburg, Mölndal, Sweden. These included neurofilament light chain (NfL, NF‐Light, UmanDiagnostics AB), amyloid beta_42_ (Aβ_42_, INNOTEST β AMYLOID [1‐42], Fujirebio), phosphorylated tau_181_ (p‐tau_181_, INNOTEST PHOSPHO‐TAU [181P]; Fujirebio), and total tau (t‐tau, INNOTEST hTAU Ag, Fujirebio). The Meso Scale Discovery platform was also used to measure both CSF Aβ_42_ and CSF Aβ_40_ (MSD MULTI‐SPOT Human [6E10] Aβ Triplex Assay) in a subset of patients (NYPUM, *n* = 69), for which the amyloid positivity cutoff for the Aβ_42/40_ ratio is < 0.89. All samples were blinded prior to analysis.

### 2.4 Histological staining

Histology and neuropathological diagnoses were made blinded to αSyn‐SAA results. *Post mortem* tissue was collected from the brain, brainstem, and cerebellum as well as from cervical, thoracic, and lumbar regions of the spinal cord of PD patients (*n* = 8) and PSP patients (*n* = 3) and immersion fixed in 4% paraformaldehyde in 0.1 M Na phosphate, pH 7.4, at room temperature. Paraffin‐embedded sections (4 µm) were stained with hematoxylin and eosin. Immunohistochemical staining was performed with antibodies against p62, tau (AT8), Aβ, and αSyn according to the manufacturer's recommendations using the ES system and ES reagents (Ventana Medical Systems Inc.). Biotin‐conjugated secondary antibodies coupled to an avidin‐horseradish peroxidase conjugate 3‐amino‐9‐ethylcarbazole (brown color) was used. Sections were counterstained with hematoxylin, washed, and mounted with Glycergel Mounting Medium (DakoCytomation). Autopsy diagnoses were consistent with clinical diagnoses and were established before the αSyn‐SAA study started. However, there were two exceptions: patients 6 and 14. In these two patients, evaluation of the brain pathology was done by a blinded investigator after disease‐incongruent αSyn‐SAA results had been obtained, to shed further light on αSyn‐SAA negative PD and αSyn‐SAA positive PSP. These results are discussed but were not used for estimating αSyn‐SAA sensitivity/specificity. All antibodies used can be found in Table S2 in supporting information.

### 2.5 αSyn‐SAA

#### 2.5.1 Gain adjustment

Prior to the assay, gain adjustment calibration of the FLUOstar Ω Reader (BMG Lab Technologies) was performed using Atto 425 (Millipore Sigma).^43^ Briefly, a calibration curve, which included a calibration standard at 3.66 µM, was prepared using Atto 425 diluted in DMSO at different concentrations and pipetted onto a black 96‐well clear‐bottom Nunc microplate (ThermoFisher Scientific). The plate was sealed with Applied Biosystems MicroAmp Optical Adhesive Film (ThermoFisher Scientific) and loaded onto the FLUOstar Ω Reader (BMG Lab Technologies). The calibration point was measured at 67% gain using filters 448‐10/482‐10 and gain was adjusted so that the calibration standard corresponded to 200,000 RFU. After gain adjustment, calibration curve was measured, and values were plotted and fitted to a straight line (*R* ^2^ > 0.9).

#### 2.5.2 Amplification assay

The αSyn‐SAA was implemented in house and performed according to Ma et al.,^43^ with several modifications. Briefly, two 1/8‘’ silicon nitride (Si3N4) beads, previously washed with nuclease‐free water (Millipore Sigma), were added to each well of a black 96‐well clear‐bottom Nunc microplate (ThermoFisher Scientific). All CSF samples were vortexed and 40 µL CSF sample, as well as negative and positive controls, was added to each well. All samples were run in triplicate. Positive control consisted of a PD sample, while the negative control was synthetic CSF (sCSF; Amprion Inc.). Next, 60 µL of the substrate mixture was added to the wells and consisted of recombinant monomeric αSyn with a C‐terminal histag at 0.3 mg/mL (Amprion Inc.), 100 mM 1,4‐piperazinediethanesulfonic acid (PIPES), pH 6.5 (Millipore Sigma), 440 mM NaCl (Lonza), 0.1% sodium lauroyl sarcosinate (Millipore Sigma), and 10 µM ThT (Millipore Sigma). The plate was sealed with Applied Biosystems MicroAmp Optical Adhesive Film (ThermoFisher Scientific) and loaded onto the FLUOstar Ω Reader (BMG Lab Technologies). The samples were then subjected to 97 agitation cycles which consisted of 1 minute at 800 rpm, followed by 14 minutes without shaking at 37°C, for 24 hours. Fluorescence was measured using 448 nm for excitation and 482 nm for emission. Data were recorded in the Omega software (5.70 R2, BMG Lab Technologies). The different outcomes of the αSyn‐SAA are described in Table S3 in supporting information. Samples that yielded an inconclusive outcome (6% of all samples) were excluded. All analyses were conducted blinded at the Neurochemistry Laboratory, in the Sahlgrenska University Hospital, in Mölndal, Sweden.

### 2.6 Statistical analysis

Assay performance for different disease groups was determined using confusion matrixes, Fisher exact test, and receiver operating characteristic (ROC) curves, which generated an area under the curve (AUC). Group‐wise comparisons were assessed by Mann–Whitney *U* or Kruskal–Wallis test followed by Dunn post hoc test for continuous variables, and by linear regression models for continuous variables and logistic regression models for binary variables adjusted for age and sex as confounders. Linear regression models were also used to determine associations between clinical features and SAA outcome and signal intensity. Kaplan–Meier survival curves were used to assess time until dementia diagnosis or exit from study. The number of patients available for follow‐up analysis decreased with time due to their death, which was determined to not significantly change the age distribution in the respective groups. Statistical significance was set at *P *< 0.05. All statistical analyses were performed in the R (v 4.3.2) statistical environment.^44^

## 3 RESULTS

### 3.1 αSyn‐SAA performance

The αSyn‐SAA successfully detected αSyn seeds in 90% of the PD samples and 81% of the MSA samples, while also identifying all HC as negative except for one participant, yielding high sensitivity and specificity (Table 1). The HC participant positive for αSyn was followed for 8 years without clinical or radiological evidence of any neurodegenerative disease, having a normal DATSCAN on four consecutive follow‐ups. When grouping synucleinopathies (PD and MSA) together, the assay collectively identified αSyn seeds in 88% of all patients, while exhibiting 97% specificity against healthy individuals, and 67% against patients with tauopathies (PSP and CBS). Most PSP patients were negative for the presence of αSyn seeds (60%), while all CBS patients were negative, with a total of 67% negative CSF samples in the tauopathy group.

**TABLE 1:** Performance of the αSyn‐SAA in the studied diagnostic groups.

Group comparison	Sensitivity	Specificity
HC vs. PD	0.90 (0.82–0.95)	0.97 (0.82–1.00)
HC vs. MSA	0.81 (0.61–0.93)	0.97 (0.82–1.00)
HC vs. synucleinopathies	0.88 (0.81–0.93)	0.97 (0.82–1.00)
Tauopathies vs. synucleinopathies	0.88 (0.81–0.93)	0.67 (0.43–0.85)

*Note*: Synucleinopathies include PD and MSA patients and tauopathies include PSP and CBS patients.

Abbreviations: αSyn‐SAA, α‐synuclein seed amplification assay; CBS, corticobasal syndrome; HC, healthy, age‐matched controls; MSA, multiple system atrophy; PD, Parkinson's disease; PSP, progressive supranuclear palsy.

**FIGURE 1:**
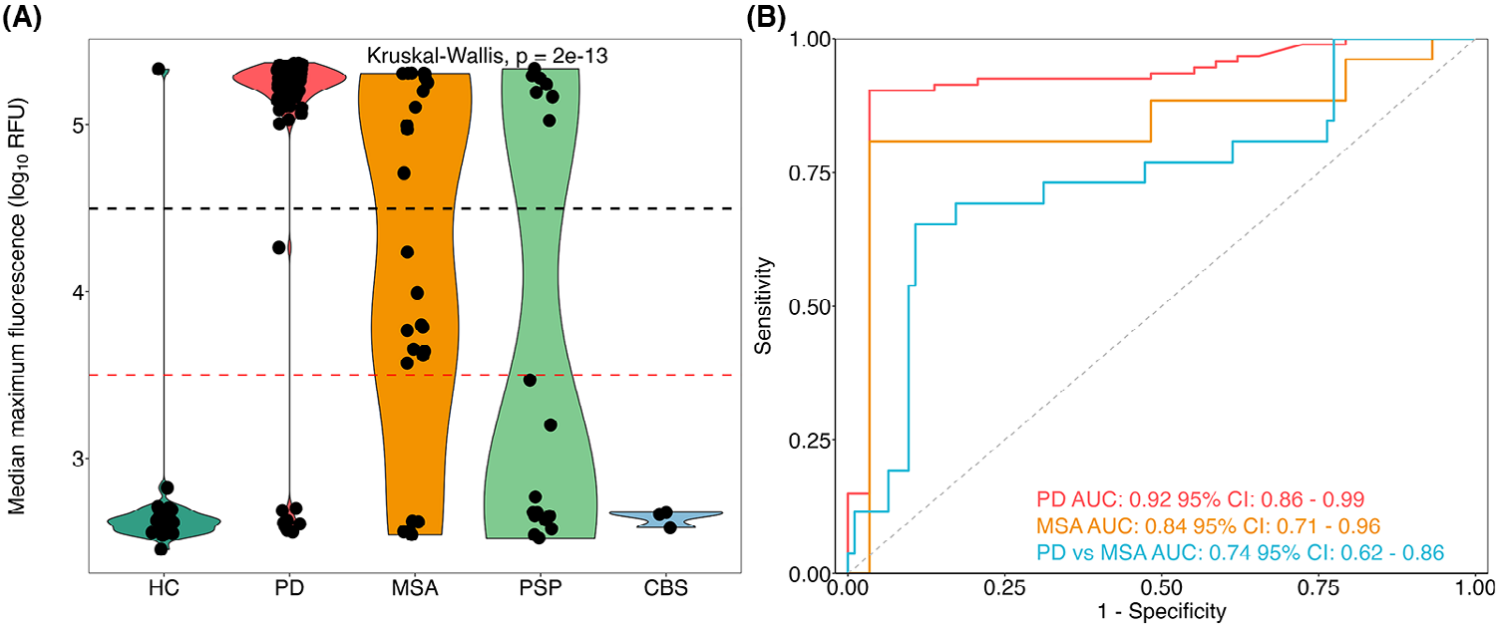
Diagnostic performance of the optimized αSyn‐SAA. A, Median maximum fluorescence (Fmax) of three replicates for each sample within the diagnostic groups. Red dashed line represents positivity limit (RFU = 3000), black dashed line represents threshold for Type‐1 seed (RFU = 45,000). B, ROC curve discriminating PD from HC, MSA from HC, and MSA from PD based on Fmax of three replicates for each sample. Fmax was log‐transformed to aid visualization. αSyn‐SAA, α‐synuclein seed amplification assay; AUC, area under the curve; CBS, corticobasal syndrome; CI, confidence interval; HC, healthy control; MSA, multiple system atrophy; PD, Parkinson's disease; PSP, progressive supranuclear palsy; RFU, relative fluorescence unit; ROC, receiver operating characteristic.

Median maximum fluorescence (Fmax) of all replicates for each sample can be found in Figure 1A, in which the difference in αSyn positivity across diagnostic groups is shown (*P *= 2e‐13, Kruskal–Wallis). The αSyn‐SAA was able to moderately discriminate between PD and MSA based on Fmax (AUC = 0.74, 95% confidence interval [CI] 0.62–0.86), which also discriminated PD and MSA from HC (AUC = 0.92, 95% CI 0.86–0.99 and AUC = 0.84, 95% CI 0.71–0.96, respectively; Figure 1B).

### 3.2 αSyn‐negative Parkinson's disease patients show an atypical clinical phenotype

Next, we assessed clinical features in the context of SAA outcome to characterize patients based on αSyn status. Clinical data for all patients stratified as SAA+ or SAA– can be found in Table 2.

**TABLE 2 alz70284-tbl-0002:** Clinical data for participants at inclusion in the study.

	HC SAA+ (*n* = 1)	HC SAA– (*n* = 28)	PD SAA+ (*n* = 84)	PD SAA– (*n* = 9)	*P*	MSA SAA+ (*n* = 21)	MSA SAA– (*n* = 5)	*P*	PSP SAA+ (*n* = 7)	PSP SAA– (*n* = 11)	*P*	CBS SAA– (*n* = 3)
Age (years)	65.6	69.0 (5.7)	67.7 (7.9)	74.3 (3.8)	**0.02**	66.9 (9.6)	66.1 (8.3)	ns	75.9 (4.8)	72.1 (5.0)	ns	61.89 (6.89)
Sex (M/F)	0/1	16/12	49/33	6/3	ns	13/8	2/3	ns	3/4	6/5	ns	2/1
Symptom duration (months)	–	–	20.5 (16)	21.6 (16.9)	ns	21 (10.7)	25.8 (10.8)	ns	18.9 (16.2)	27.9 (31.5)	ns	36 (16.9)
UPDRS total, baseline	–	–	34 (12)	42 (12.7)	ns	37.6 (17.3)	22	ns	42.2 (10.2)	32.4 (11.3)	**0.03**	53
**Motor**												
UPDRS III, baseline	–	–	24.3 (10.3)	29.7 (10.4)	ns	26.6 (12.2)	37.5 (36)	ns	31 (7)	22.8 (10.9)	ns	35
H&Y stage, baseline	–	–	2 (0.5)	2.6 (0.6)	**0.003**	2.6 (1)	2.7 (1.5)	ns	2.5 (0.5)	2.5 (0.6)	ns	1.5 (0.7)
PIGD score, baseline	–	–	0.4 (0.2)	1 (0.5)	**0.002**	0.9 (0.8)	–	–	1 (0.3)	0.4 (0.4)	**0.03**	–
Tremor score, baseline	–	–	0.5 (0.3)	0.1 (0.1)	**0.002**	0.3 (0.1)	–	–	0.1 (0.1)	0.1 (0.2)	ns	–
**Cognition**												
MMSE, baseline	30	29 (0.8)	28.6 (1.3)	28.2 (1.3)	ns	28.3 (2.1)	29	–	27.1 (1.9)	27.4 (2.7)	ns	25 (7)
Cognition (total *n*)	1	0	65	9	–	0	1	–	0	0	–	0
Normal, *n* (%)	1 (100)	–	40 (62)	3 (34)	ns	–	–	–	–	–	–	–
MCI, *n* (%)	–	–	25 (38)	6 (66)	ns	–	1 (100)	–	–	–	–	–
**Ancillary investigations**												
Olfaction, score at baseline	11	9.3 (2)	6.4 (2.4)	7.8 (3.4)	ns	7.6 (3)	–	–	6.5 (2.8)	8 (2)	ns	–
DATSCAN, abnormal/*n* (%)	0/1 (0)	0/28 (0)	64/64 (100)	8/9 (88)	–	9/9 (100)	5/5 (100)	–	7/7 (100)	11/11 (100)	–	–
REM SBD, yes/no (% yes)	–	–	9/57 (13)	0/7 (0)	ns	2/7 (22)	–	–	0/6 (0)	0/6 (0)	–	–
Smoker, yes/no (% yes)	–	–	28/54 (24)	5/4 (55)	ns	6/3 (66)	–	–	0/6 (0)	1/4 (20)	ns	–
Blood pressure drop (supine – standing 3 min)	–	–	−5.9 (16)	−7.2 (16.9)	ns	−27.3 (18.1)	−	−	−4.6 (12.8)	−3.6 (12.6)	ns	−

*Notes*: Individuals were classified as SAA+ or SAA–, according to αSyn‐SAA outcome. Continuous data are presented as mean (standard deviation) and binary data are expressed in counts and percentages. Extensive clinical data and deep phenotype characterization, e.g., full neuropsychological test battery results, were not available for all patients. Olfaction was investigated by Brief Smell Inventory Test (BSIT). P values adjusted for age and sex are available in Tables S4 and S5 in supporting information. Bold values denote significant *P* values.

Abbreviations: αSyn‐SAA, α‐synuclein seed amplification assay; H&Y, Hoehn & Yahr; MCI, mild cognitive impairment; MMSE, Mini‐Mental State Examination; MSA, multiple system atrophy; PD, Parkinson's disease; PIGD, postural instability and gait difficulty; PSP, progressive supranuclear palsy; REM SBD, rapid eye movement sleep behavior disorder (evaluated by interview); UPDRS, Unified Parkinson's Disease Rating Scale.

We observed that SAA– PD patients have slightly more advanced motor symptoms at baseline, compared to SAA+ PD patients (*P *< 0.01), as assessed by UPDRS H&Y (Table 2). This difference partially reflected a motor phenotype characterized by postural instability and gait difficulties. SAA+ PD patients exhibit more tremor (*P *< 0.01; Table 2, Table S4 in supporting information) compared to SAA– PD patients at baseline. Conversely, SAA– PD patients have higher PIGD scores (*P *< 0.01; Table 2, Table S4), and all presented with a PIGD phenotype at baseline (Table 3). No SAA– PD patient showed prominent resting tremor, although some tremor was found among these patients.

**TABLE 3:** Clinical and molecular characteristics of patients with unexpected SAA outcome.

Clinical diagnosis in life	Patient	SAA	Autopsy diagnosis	Other pathology at autopsy	Presenting symptoms	Disease phenotype	Cognitive function	Diagnostic red flags	DATSCAN	CSF Aβ_42_	UKPDSBB criteria	MDS PD criteria
**PD**	Patient 1	Negative	NA	NA	Bradykinesia, postural instability	PIGD	Normal	Brief Levomepromazin use 15 years prior to baseline; postural instability; symmetric parkinsonism	Normal	Low	PD	No
	Patient 2	Negative	NA	NA	Bradykinesia	PIGD	Normal	Symmetric parkinsonism; Achilles reflex lapse	Pathological	Low	PD	Yes
	Patient 3	Negative	NA	NA	Gait disturbance, mild dysphagia,	PIGD	MCI	Mild kinetic tremor; palpebral tremor; symmetric parkinsonism	Pathological	Low	PD	Yes
	Patient 4	Negative	NA	NA	Bradykinesia, reduced arm swing	PIGD	Normal	Urgency incontinence; symmetric parkinsonism	Pathological	Low	PD	Yes
	Patient 5	Negative	NA	NA	Bradykinesia, shuffling steps	PIGD	MCI	Repeated falls at baseline; postural instability; symmetric parkinsonism	Pathological	Low	PD	Yes
	Patient 6	Negative	AD‐PSP	No LB; Presence of Aβ deposits, p‐tau and Tau‐4R inclusions	Resting tremor, bradykinesia, micrographia	PIGD	MCI	−	Pathological	Low	PD	Yes
	Patient 7	Negative	NA	NA	Bradykinesia, propulsion	PIGD	MCI	Symmetric parkinsonism	Pathological	Low	PD	Yes
	Patient 8	Negative	NA	NA	Gait disturbance, hyposmia	PIGD	MCI	Mildly slow oculomotor saccades	Pathological	Low	PD	Yes
	Patient 9	Negative	NA	NA	Stiffness during gait, bradykinesia	PIGD	MCI	Mild kinetic tremor	Pathological	Low	PD	Yes
**Atypical features**												
**PSP**	Patient 10	Positive	NA	NA	Difficulty with voluntary eye movements	RS	−	−	Pathological	Low	−	−
	Patient 11	Positive	NA	NA	Bradykinesia	RS	MCI	Early postural instability; sleep apnea; symmetric parkinsonism	Pathological	Low	−	−
	Patient 12	Positive	NA	NA	Bradykinesia	PSP‐P	Normal	Symmetric parkinsonism	Pathological	Low	−	−
	Patient 13	Positive	NA	NA	Bradykinesia	RS	Normal	Polyneuropathy signs; ataxia; dopa‐related dyskinesia; ocular palsy; early postural instability	Pathological	Low	−	−
	Patient 14	Positive	AD‐PD	Neocortical LB spread; AD pathology	Bradykinesia	RS	MCI	Early postural instability; symmetric parkinsonism	Pathological	Normal	−	−
	Patient 15	Positive	NA	NA	Bradykinesia	PSP‐F	MCI	Symmetric parkinsonism; early cognitive impairment	Pathological	Low	−	−
	Patient 16	Positive	PSP	NA	Bradykinesia	RS	MCI	Symmetric parkinsonism; early cognitive impairment	Pathological	Low	−	−

*Notes*: Clinical and molecular characteristics of αSyn‐SAA– PD and SAA+ PSP patients, where the SAA disease‐incongruent result raise suspicion of different or comorbid disease pathology. CSF Aβ_42_ level, as measured by INNOTEST β AMYLOID (1‐42), was deemed “Low” if below the median and interquartile range of control group (868 [716,1009] pg/mL) and “Normal” if equal or above.

Abbreviations: αSyn‐SAA, α‐synuclein seed amplification assay; Aβ, amyloid beta; CSF, cerebrospinal fluid; MCI, mild cognitive impairment; MDS, Movement Disorder Society; NA, non‐applicable; PD, Parkinson's disease; PIGD, postural instability and gait difficulty; PSP‐F, PSP with predominant frontal presentation; PSP‐P, PSP‐Parkinsonism; p‐tau, phosphorylated tau; RS, Richardson syndrome subtype of PSP; UKPDSBB, UK Parkinson's Disease Society Brain Bank.

**FIGURE 2 alz70284-fig-0002:**
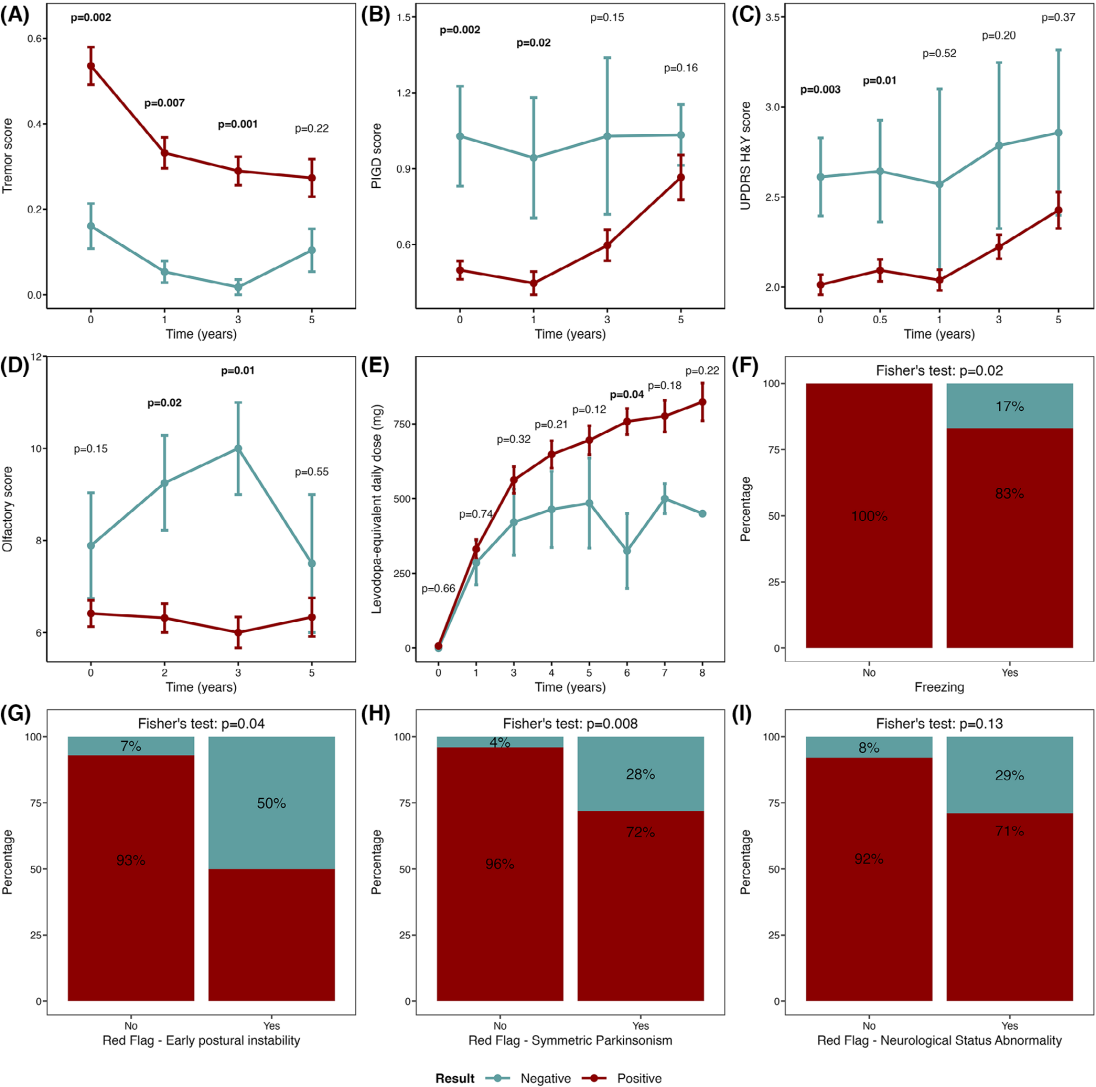
Performance of SAA+ and SAA– PD patients on clinical examination. These included longitudinal scores for (A) tremor, (B) PIGD, (C) UPDRS Hoehn & Yahr stage, (D) olfactory test, and (E) levodopa‐equivalent daily dose (mg). (F) Percentage of PD patients with and without freezing, (G) diagnostic red flag early postural instability, (H) diagnostic red flag symmetric parkinsonism, and (I) diagnostic red flag other neurological status abnormality. The panels F‐I were investigated at baseline. Points and error bars represent mean + standard error of the mean. Mann–Whitney *U* test was performed between SAA– and SAA+ PD patients for the different time points. Later time points did not have the same number of score data points due to the death of patients. *P* values adjusted for age and sex are available in Table S4 in supporting information. Bold *P* values in longitudinal graphs denote significant *P* values. Fisher exact test was used to assess difference in count/percentage proportions. H&Y, Hoehn & Yahr stage; PD, Parkinson's disease; PIGD, postural instability and gait difficulty; SAA, seed amplification assay; UPDRS, Unified Parkinson's Disease Rating Scale.

Over time, SAA+ PD patients have increased tremor scores, while SAA– generally have higher PIGD scores (Figure 2A, B), although both scores in SAA– and SAA+ PD patients converge in later disease stages. In agreement with their motor phenotype, SAA– PD patients had an increased frequency of freezing at baseline (Figure 2F). Additional analysis also showed that, compared to SAA+ PD, SAA– PD patients performed significantly worse in the TUG test, indicating impaired balance and gait (Figure S1 in supporting information). Examining H&Y staging longitudinally, we observed a general trend toward higher H&Y scores in SAA– PD compared to SAA+ PD patients, with significant differences between SAA+ and SAA– at baseline and 6 months (*P *< 0.05; Figure 2C). According to diagnostic red flags, SAA– PD patients have a higher incidence of early postural instability (*P *= 0.04), symmetric parkinsonism (*P *< 0.01), and other abnormal neurological signs (when corrected for age and sex, Table S5 in supporting information), compared to SAA+ PD patients (Figure 2F–I). Olfactory function, assessed longitudinally, showed significantly better olfaction in SAA– compared to SAA+ PD patients (Figure 2D). Levodopa‐equivalent daily dose (LEDD) differed in SAA+ and SAA– PD patients, with SAA– PD initially benefitting from dopaminergic medication, but after increased dosage eventually reaching a plateau, suggesting loss of responsiveness (Figure 2E). The difference in LEDD between SAA+ and SAA– PD was significant at 6 years after baseline. All significant *P* values remained significant after correcting for age and sex, except for LEDD (Table S4).

Given the disease‐incongruent SAA outcome in SAA– PD, diagnoses were re‐evaluated based on longitudinal data and[Table alz70284-tbl-0002] available autopsies (see Table 3 and Table S6 in supporting information, in particular Patient 6). None of the SAA– PD patients were determined to have essential tremor (ET), normal pressure hydrocephalus, CBS, MSA, vascular parkinsonism, or a rapidly progressing dementia syndrome, for example, Creutzfeldt–Jakob disease. Table 2 shows vascular risk factors (smoking and blood pressure) and vascular parkinsonism assessment, all of which were similar in SAA– and SAA+ PD. None of the SAA– PD patients were determined to fulfil diagnostic criteria for probable PSP, and none of the patients had a clinical PSP diagnosis. However, when re‐evaluated, AD, PSP–AD comorbidity, and non‐typical PSP‐Parkinsonism (PSP‐P) syndromes were determined reasonable alternative diagnoses in several cases. Findings supporting AD with motor symptoms are reported in the next section.

### 3.3 AD brain pathology, lower CSF Aβ_42_, and MCI in αSyn– PD patients

Pathologically confirmed diagnosis was available for eleven patients, of whom seven were PD SAA+ and one was PD SAA–, with the other three being two SAA+ PSP and one SAA– PSP patients (Table S6). Almost all cases of autopsy‐confirmed PD corresponded to their clinical diagnosis as well as SAA outcome (Table S6).

As stated, and shown in Table 2, SAA– PD patients presented with symptoms at baseline characteristic of a PIGD phenotype (postural instability, decreased arm swing, shuffling steps, gait disturbance, stiffness), and most of the SAA– PD patients also had MCI (67%). All except one (Patient 1) had a pathological DATSCAN, indicating degeneration in the nigrostriatal system. Indeed, Patient 1 was the only patient who did not fulfil a clinical PD diagnosis when re‐evaluated according to the most recent MDS diagnostic criteria.^34^ Several of the SAA– PD patients displayed subtle diagnostic red flags, which were, however, determined so mild that they did not exclude a PD diagnosis.

**FIGURE 3:**
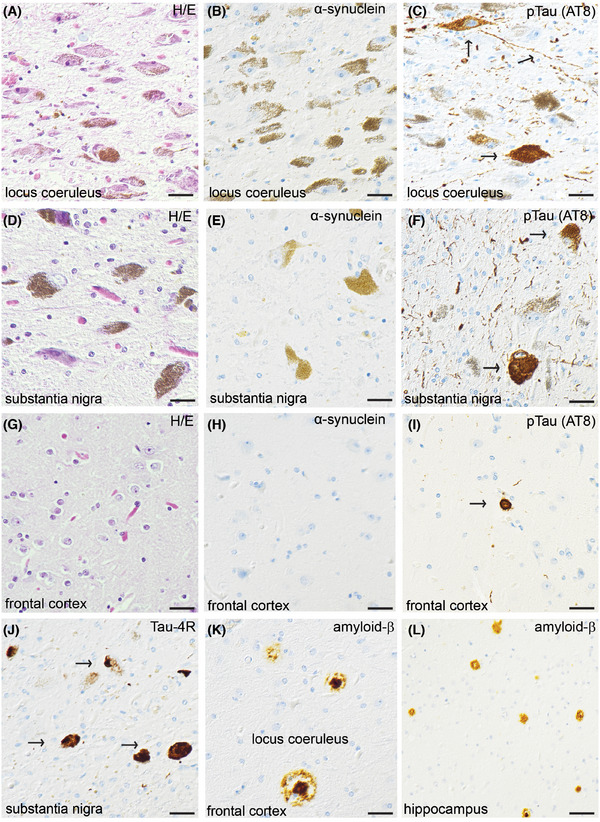
Pathoanatomical diagnosis and immunohistochemistry in an αSyn negative PD patient. Histology included hematoxylin and eosin (H/E) staining in the (A) locus coeruleus, (D) substantia nigra, and (G) frontal cortex; αSyn staining in the (B) locus coeruleus, (E) substantia nigra, and (H) frontal cortex; p‐tau (AT8) staining in the (C) locus coeruleus, (F) substantia nigra, and (I) frontal cortex; Tau‐4R staining in the (J) substantia nigra; and Aβ staining in the (K) frontal cortex, and (L) hippocampus. Aβ, amyloid beta; PD, Parkinsons’ disease; p‐tau, phosphorylated tau.

The SAA– PD patient (Patient 6) who died and underwent *post mortem* examination had no LBs in substantia nigra or elsewhere (Table S6). The same patient showed extensive cortical tau and amyloid deposits, consistent with Braak stage IV to V and[Fig alz70284-fig-0002] Thal stage 4, respectively, consistent with comorbid, advanced AD and PSP (Figure 3).

**FIGURE 4:**
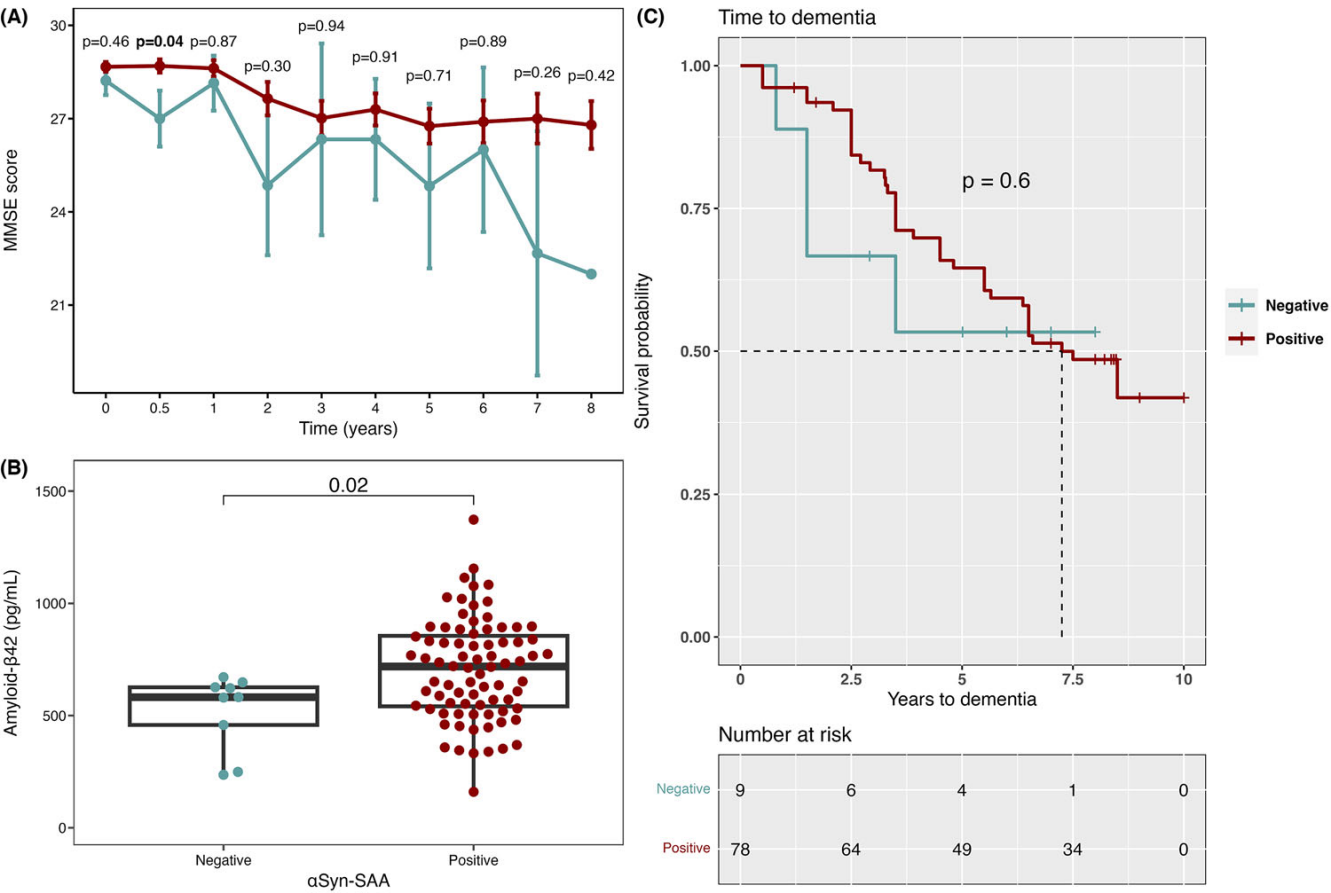
αSyn status and cognition in PD patients. A, Longitudinal MMSE score for SAA– and SAA+ PD patients. B, CSF amyloid‐β_42_ (Aβ_42_) levels in SAA– and SAA+ PD patients. C, Kaplan–Meier survival curve for time until dementia diagnosis for SAA– and SAA+ PD patients, *P* value was obtained using the log‐rank test to compare survival distribution of both groups. Later time points did not have the same number of MMSE score data points due to the death of many patients. Vertical dashes along survival curves represent event (dementia or exit from study). Points and error bars in longitudinal data represent mean + standard error of the mean. Mann–Whitney *U* test was used to compare SAA– and SAA+ PD patients. Bold denotes significant *P* values. *P* values adjusted for age and sex for MMSE scores and levels of Aβ_42_ are available in Tables S4 and S5 in supporting information. αSyn, α‐synuclein; Aβ, amyloid beta; CSF, cerebrospinal fluid; MMSE, Mini‐Mental State Examination; PD, Parkinsons’ disease; SAA, seed amplification assay.

Aβ_42_ levels in CSF were decreased in SAA– PD compared to SAA+ PD (*P *= 0.02; Figure 4B), consistent with the high prevalence of MCI and with the pronounced amyloid inclusions found in the brain of Patient 6. This decrease was consistent across both Aβ_42_ analyses (INNOTEST β AMYLOID [1‐42] and MSD Aβ Triplex Assay; Figure S2A in supporting information). Furthermore, all patients with SAA– PD had apparently low CSF Aβ_42_ compared to HC (Table 3) and the Aβ_42/40_ ratio, which was available for a subset of patients (*n* = 69), was significantly lower in SAA– PD patients (of whom five out of seven were amyloid‐positive according to the MSD assay), compared to both SAA+ PD and HC (*P *= 0.008; Figure S2B). Together with the clinical phenotype, these findings support the presence of AD in these patients, in isolation or combined with comorbid tauopathy.

Considering this AD–PD overlap and the high prevalence of MCI in αSyn‐SAA negative PD, detailed cognitive testing, specifically for episodic memory and language, were assessed. MMSE scores were generally lower in SAA– PD patients, with MMSE score at 6 months being significantly lower in SAA– PD compared to SAA+ (*P *< 0.05; Figure 4A). The rate of dementia development was similar in SAA– PD and SAA+ PD patients, however with a trend toward shorter time‐to‐dementia in SAA– PD (Figure 4C).

Episodic memory was significantly worse in SAA– PD compared to SAA+ PD, with lower test scores at baseline (*P *= 0.03; Table S7 in supporting information). No association between SAA and language was observed (Table S7). All significant *P* values, except for episodic memory, remained significant after correcting for age and sex (Table S7).

### 3.4 αSyn seeding correlates with dopamine transporter uptake deficits

**FIGURE 5:**
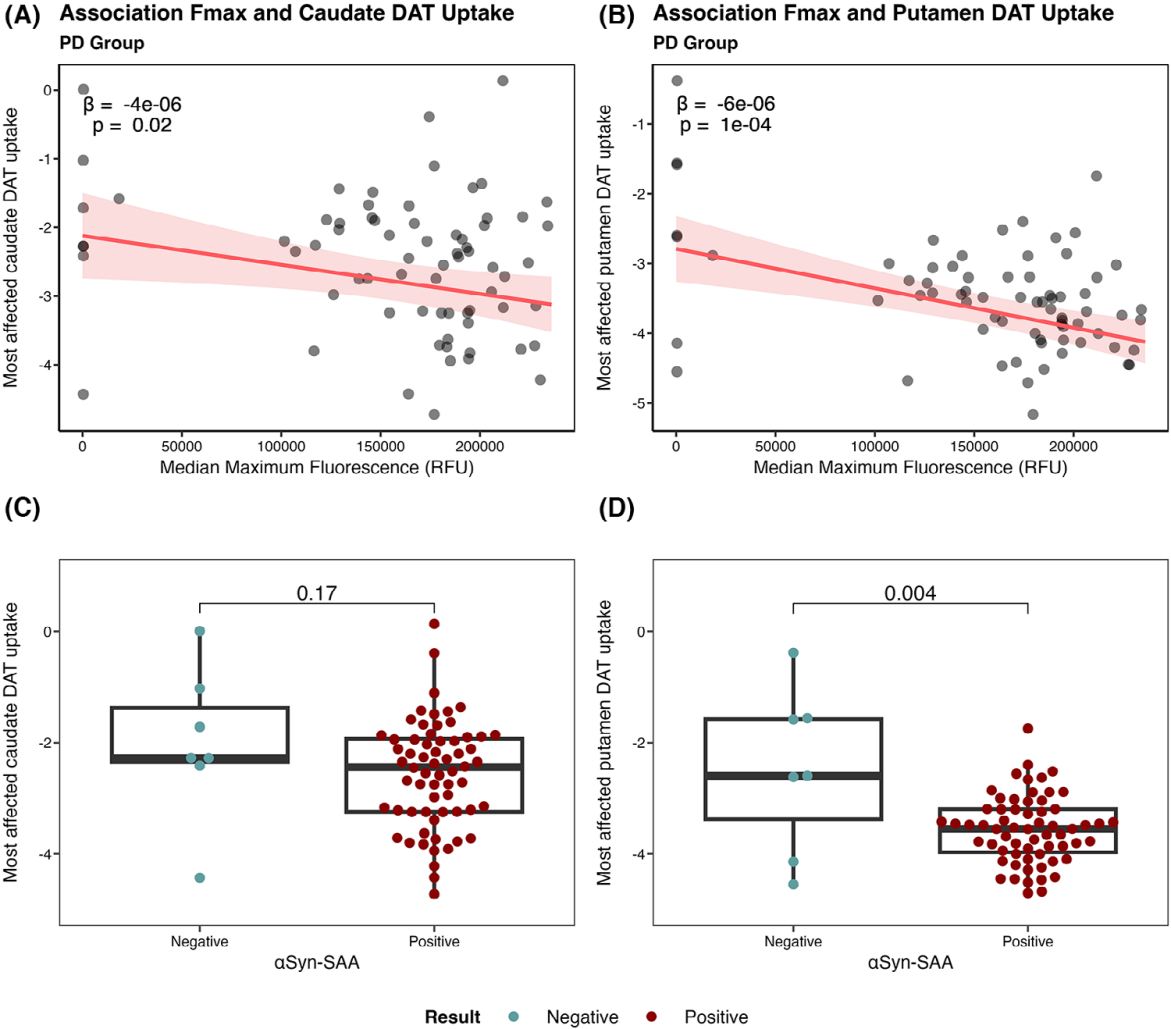
αSyn status and DAT uptake in the PD group. A, Association of caudate DAT uptake and median maximum fluorescence. B, Association between putamen DAT uptake and median maximum fluorescence. C, Caudate DAT uptake in SAA– and SAA+ PD patients. D, Putamen DAT uptake in SAA– and SAA+ PD patients. Some PD patients did not have DATSCAN data available. Linear models were used to perform the comparison between SAA– and SAA+ PD patients. *P* values adjusted for age and sex for levels of DAT uptake in caudate and putamen are available in Table S5 in supporting information. Bold denotes significant *P* values. αSyn, α‐synuclein; DAT, dopamine transporter; PD, Parkinson's disease; RFU, relative fluorescence unit; SAA, seed amplification assay.

Next, PD patients were compared with regard to presynaptic dopaminergic integrity as measured by DATSCAN imaging (Figure 5). To determine whether DAT uptake and αSyn‐SAA signal intensity were linked, we assessed the association of most affected caudate and putamen DAT uptake and Fmax.

Both the caudate and putamen DAT uptake is associated with Fmax in the PD group (β = –4e‐06, *P *= 0.02 and β = –6e‐06, *P *= 1e‐04, respectively; Figure 5A and B). DAT uptake for both brain structures was also assessed between SAA– and SAA+ PD patients (Figure 5C and D). While there was no difference between SAA– and SAA+ PD individuals in the caudate, DAT uptake in the putamen was most decreased in SAA+ patients (Figure 5D). All *P* values remained significant after correcting for age and sex (Table S5). Repeating the analysis with only SAA+ PD cases, the association between SAA Fmax and putamen DAT uptake remained significant (data not shown).

### 3.5 Alpha‐synucleinopathy and lower CSF Aβ_42_ in some patients with PSP

While more than half of PSP patients were negative for the presence of αSyn, according to the αSyn‐SAA, a substantial subset of PSP patients (39%) had positive SAA (Table 2). SAA+ PSP patients showed increased disease severity based on UPDRS total score (*P *= 0.03), exhibiting higher PIGD scores (*P *= 0.03), and displayed lower levels of CSF NfL (*P *= 0.04), compared to SAA– PSP patients (Table 2, Figure S3 in supporting information). However, these comparisons lost significance once adjusted for age and sex (Tables S4, S5). Lower levels of Aβ_42_ were observed in SAA+ PSP compared to SAA– PSP, even after adjusting for confounders (Figure S3, Table S5).

Almost all SAA+ PSP had a Richardson's syndrome (RS) phenotype, which is consistent with bradykinesia as a presenting symptom, as well as the presence of early postural instability and symmetric parkinsonism (Table 3). Approximately 67% of SAA+ PSP patients also had MCI, and all had abnormal nigrostriatal imaging (as investigated by DATSCAN). The atypical features were consistent with a PSP diagnosis (Table 3).

Only three PSP patients underwent *post mortem* confirmation (Table S6), with heterogenous results. Patient 16 was confirmed PSP at autopsy while having a positive SAA result, indicating a comorbid synucleinopathy (such as PD) together with the expected 4‐repeat tauopathy. The *post mortem* examination of the second SAA+ PSP patient (Patient 14) showed comorbid AD and PD, without PSP pathology, manifested by advanced neocortical αSyn as well as amyloid deposits. The remaining PSP patient (Patient 24) had no aggregated αSyn at SAA (being SAA–) and was confirmed as PSP at autopsy.

## 4 DISCUSSION

In this study, we implemented a modified version of an optimized αSyn‐SAA reported by Ma et al.,^43^ and used it to stratify patients according to underlying αSyn pathology in a mostly population‐based setting. Thereby, we could characterize patients with PD, as well as with MSA and PSP, based on their αSyn‐SAA outcome, exploring the clinical and molecular relevance of αSyn pathology in these patients. Particularly, we characterized PD patients negative for αSyn, whose clinical presentation and laboratory investigations were consistent with an AD‐like syndrome, and, in some cases, with complex neurodegeneration, involving both PSP‐type tauopathy and AD.

The αSyn‐SAA demonstrated high diagnostic performance for PD, detecting αSyn seeds in 90% of individuals, similar to previous studies.^25^, ^27^, ^29^ In MSA, the αSyn‐SAA detected αSyn seeds in 80% of patients. Overall, the αSyn‐SAA exhibited high accuracy for the detection of synucleinopathies (especially PD). The lower αSyn positivity in MSA could be due to the αSyn‐SAA being less sensitive for early MSA compared to early PD. Another explanation, as the clinical diagnosis of MSA is prone to misdiagnosis,^45^ is that a minority of these patients had another disease, despite fulfilling diagnostic criteria for MSA and having a pathological DATSCAN. The AUC for discriminating between PD and MSA was moderate (AUC = 0.74, 95% CI: 0.62–0.86), indicating that αSyn‐SAA fluorescence levels might be the best way to distinguish these synucleinopathies, as previously observed.^26^, ^43^ The association between DAT uptake and Fmax strengthens the link between αSyn seeds in CSF and PD pathology. This suggests that the CSF αSyn‐seeding measurements can reflect the severity of nigrostriatal degeneration, which were generally more severe for SAA+ PD patients. The outcome for the non‐synucleinopathy group (PSP/CBS) was mostly negative, but with limited specificity due to the positive cases in PSP patients.

Longitudinally, the clinical progression of PD and PSP patients was investigated based on αSyn aggregation status, particularly in SAA– PD and SAA+ PSP, while the lack of follow‐up data and early deaths of MSA patients did not allow for a comprehensive analysis.

In PSP patients, SAA+ PSP trended toward increased PIGD scores and disease severity, which may be expected if they have comorbid PD together with the expected 4‐repeat tauopathy of PSP.^46^ Furthermore, lower NfL in SAA+ PSP patients might suggest a PD‐like phenotype, or PD with oculomotor deficits misdiagnosed as PSP, because PD patients generally have lower NfL than PSP patients.^47^ Confounding the diagnosis, the combination of AD and PD pathology could possibly cause a clinical PSP‐like syndrome, such as demonstrated by Patient 14. However, recent studies report αSyn aggregation (in LBs) in the brains of pathologically confirmed PSP patients.^13^, ^48^ This suggests a positive SAA may be a true indication of comorbid αSyn aggregation in PSP. Co‐occurring αSyn aggregation in PSP is consistent with the other PSP patients who were pathologically confirmed at *post mortem* examination in this study, one of whom was SAA+.

In PD, SAA– and SAA+ PD patients presented with distinctly different phenotypes and progression, especially regarding motor phenotype, olfactory function, and CSF Aβ_42_ levels. Together with differences in striatal DAT imaging, exhibiting a more normal putaminal uptake, these characteristics imply that αSyn‐SAA negative PD patients are not false negatives. While SAA– PD patients were slightly older and presented with a pronounced PIGD phenotype, with no/mild tremor and less sustained benefit to dopaminergic medication, SAA+ PD showed the classical PD resting tremor to a larger extent.^49^ H&Y score was generally higher in SAA– than SAA+ PD patients, emphasizing that a negative αSyn‐SAA is associated with a more advanced PD stage, and making alternative diagnoses, like ET, unlikely. Additionally, increased freezing reflects a different type of motor impairment in SAA– PD patients. Performance in the olfactory test, one of the earliest indicators of PD,^50^, ^51^ showed that SAA– PD patients had better olfaction over time than in SAA+ PD. Similar findings regarding olfaction in SAA– PD were observed in the Parkinson's Progression Markers Initiative cohort.^52^

In this mostly population‐based cohort, almost 70% of SAA– PD patients had MCI at baseline (compared to only 38% of SAA+ PD), which is higher than the estimated prevalence of MCI in PD in other studies.^53^ Specifically, SAA– patients with PD had worse episodic memory performance compared to other PD patients, despite not showing a clear increase in dementia incidence. Generally, compared to SAA+ PD, SAA– PD patients trended toward lower MMSE score and, importantly, showed decreased CSF Aβ_42_ levels using two different assays, and lower Aβ_42/40_ ratio. This indicates an increased risk of cognitive impairment,^54^, ^55^ likely reflecting amyloid plaque burden.^56^

The phenotypical difference between SAA– and SAA+ PD and the autopsy of Patient 6 indicate that SAA– PD patients may include misdiagnosed PSP, which may be confounded by comorbid AD. This interpretation is strengthened by the increased incidence of diagnostic red flags (e.g., early postural instability and symmetric parkinsonism) within the SAA– PD group. Other atypical movement disorders were determined unlikely based on all clinical data.^34^

However, the PIGD‐type motor symptoms, low CSF Aβ_42_ and Aβ_42/40_, and high prevalence of MCI in SAA– PD patients at baseline, together with the fact that no patient had a clinical PSP diagnosis, suggests that SAA– PD may be explained in some cases by a more pure AD with prominent motor symptoms. Such a late‐onset, motor predominant AD may mimic PD.^16^, ^18^, ^57^ In agreement, the clinical presentation of the SAA– PD patients is consistent with reports of motor symptoms in AD, which are frequently defined as akinetic and rigid, similar to the PIGD‐dominant phenotype in the SAA– PD patients observed here.^16^, ^18^, ^57^

Also consistent with an AD–PD overlap are the recent SAA studies describing over 30% αSyn positivity in AD patients,^58^, ^59^ much higher than in HCs. This αSyn positivity, together with the incidence of parkinsonism in AD, underscore the similarities between AD and PD. Parkinsonism and αSyn positivity in AD could partially be attributed to an Aβ‐driven accumulation of misfolded αSyn.^60^ Because dopaminergic neurons are one of the most vulnerable neurons, such interaction may promote dopamine deficiency and motor symptoms in AD.^61^ However, αSyn‐independent mechanisms are also reported to cause parkinsonism in AD.^62^, ^63^, ^64^ AD patients with extrapyramidal signs might not have any evidence of αSyn, and still display neuronal loss in the substantia nigra linked to tau pathology, resulting in motor symptoms.^62^, ^63^, ^65^, ^66^ Such motor symptoms may even be responsive to levodopa.^21^, ^63^, ^67^ These mechanisms would make patients with pure AD present similar to late‐onset PD, in particular in patients with MCI. Indeed, AD with parkinsonism is sometimes misdiagnosed as PD.^63^, ^64^, ^68^ In autopsy studies, 2% to 6% of clinically diagnosed patients with PD were misdiagnosed and had only AD instead of PD, while ≈ 18% had both AD and PD, which are figures that may vary with sample population selection.^68^

The high frequency of AD comorbidity found in PD and PSP patients is not surprising, given its high incidence in the general population. The mixed pathology of PD, AD, and PSP found in the SAA‐incongruent patients described here might also suggest synergistic effects of combined brain pathology. Evidence suggests that concomitant Aβ, αSyn, and tau promote the fibrillization and toxicity of one another, although the mechanism is largely elusive.^69^, ^70^, ^71^ The molecular cross‐talk between these pathologies is thought to promote more severe and complex clinical phenotypes, similar to those reported here.^70^, ^71^, ^72^, ^73^ Our findings suggest that AD pathology is a major confounder in the diagnosis of both PSP and PD. Diagnosis is likely to be especially confounded if multiple pathologies are present. Our findings highlight the αSyn‐SAA as a powerful resource for the accurate stratification of PD patients for future clinical trials. This study raises the question of whether AD‐targeted therapeutic interventions (i.e., anti‐Aβ antibodies) could be a valuable addition to the treatment of clinically diagnosed PD patients, who may have Aβ plaques, as confirmed in Patient 6 and Patient 18, especially considering the findings in SAA– PD.

Despite careful clinical, neurochemical, and neuroimaging characterization of patients and longitudinal follow‐up for up to 18 years, with several autopsies, this study is not without limitations. First, the SAA– PD group was small, limiting the power. Although Aβ_42_ levels were lower, other investigations like amyloid PET imaging and extensive autopsies to characterize SAA– PD cases were not available. Despite a substantial amount of longitudinal data, there were also fewer observations in the last time points. Finally, there was a lack of data for MSA, PSP, and CBS, because these patients died earlier in the disease course, which did not allow for a comprehensive longitudinal analysis.

In conclusion, the αSyn‐SAA is a robust assay to not only support the diagnosis of synucleinopathies, but to detect the presence of αSyn comorbidity in other parkinsonian disorders. We found that AD plays a major role in the phenotype of both clinically diagnosed PSP and PD patients with αSyn‐SAA–incongruent results. Clinically diagnosed, criteria‐compliant PD patients who test negative for αSyn are older and generally have a more severe PIGD phenotype with worse cognition, in addition to lower CSF Aβ_42_ compared to positive PD patients, which is consistent with AD traits in these patients.

## CONFLICT OF INTEREST STATEMENT

Yihua Ma, Carly M. Farris, and Luis Concha‐Marambio are Amprion employees and declare employee stock option ownership and invention of patents related to SAA assigned to Amprion. Henrik Zetterberg has served at scientific advisory boards and/or as a consultant for Abbvie, Acumen, Alector, Alzinova, ALZPath, Amylyx, Annexon, Apellis, Artery Therapeutics, AZTherapies, Cognito Therapeutics, CogRx, Denali, Eisai, LabCorp, Merry Life, Nervgen, Novo Nordisk, Optoceutics, Passage Bio, Pinteon Therapeutics, Prothena, Red Abbey Labs, reMYND, Roche, Samumed, Siemens Healthineers, Triplet Therapeutics, and Wave, has given lectures sponsored by Alzecure, BioArctic, Biogen, Cellectricon, Fujirebio, Lilly, Novo Nordisk, Roche, and WebMD, and is a co‐founder of Brain Biomarker Solutions in Gothenburg AB (BBS), part of the GU Ventures Incubator Program (outside submitted work). Kaj Blennow has served as a consultant and at advisory boards for Abbvie, AC Immune, ALZPath, AriBio, BioArctic, Biogen, Eisai, Lilly, Moleac Pte. Ltd, Neurimmune, Novartis, Ono Pharma, Prothena, Roche Diagnostics, Sanofi, and Siemens Healthineers; has served at data monitoring committees for Julius Clinical and Novartis; has given lectures, produced educational materials, and participated in educational programs for AC Immune, Biogen, Celdara Medical, Eisai, and Roche Diagnostics; and is a co‐founder of Brain Biomarker Solutions in Gothenburg AB (BBS), part of the GU Ventures Incubator Program (outside submitted work). Other authors report no disclosures. Author disclosures are available in the supporting information.

## Supporting information



Supporting Information

Supporting Information
