# Supplementary material for: Alzheimer's disease traits in Parkinson's disease without α‐synuclein seeding
Source: Alzheimers Dement. 2025 May 19;21(5):e70284. doi: 10.1002/alz.70284 (PMC12086808; doi:10.1002/alz.70284)
Supplement: Supplementary file 1 — Supporting Information [file ALZ-21-e70284-s002.docx]

**Supplementary data**

**Supplementary Table 1. Number of participants in the PD group throughout the time points assessed in this study.**

| **Longitudinal analysis** | **Years** | **Total number of individuals** |
| --- | --- | --- |
| Tremor score | 0 | 70 |
|  | 1 | 70 |
|  | 3 | 70 |
|  | 5 | 65 |
| PIGD score | 0 | 70 |
|  | 1 | 70 |
|  | 3 | 70 |
|  | 5 | 64 |
| UPDRS H&Y score | 0 | 91 |
|  | 0.5 | 72 |
|  | 1 | 73 |
|  | 3 | 72 |
|  | 5 | 68 |
| Olfactory score | 0 | 84 |
|  | 2 | 61 |
|  | 3 | 55 |
|  | 5 | 44 |
| LEDD | 0 | 73 |
|  | 1 | 73 |
|  | 3 | 72 |
|  | 4 | 69 |
|  | 5 | 66 |
|  | 6 | 53 |
|  | 7 | 49 |
|  | 8 | 34 |
| MMSE score | 0 | 87 |
|  | 0.5 | 69 |
|  | 1 | 75 |
|  | 2 | 77 |
|  | 3 | 74 |
|  | 4 | 67 |
|  | 5 | 68 |
|  | 6 | 52 |
|  | 7 | 46 |
|  | 8 | 40 |

**Supplementary Table 2. Commercially available antibodies used in this study.**

| **Description** | **Product identification** | **Manufacturer** | **Antibody dilution** |
| --- | --- | --- | --- |
| Mouse anti-human β-amyloid | M087201-2 | Agilent Technologies | 1:100 |
| Mouse anti-α-Synuclein | NCL-L-ASYN | Novocastra | 1:100 |
| Mouse anti-TAU(AT8) | BR-03 | Immunogenetics | 1:800 |
| Mouse anti-Tau 4R | 05-805 clone 1E1/A6 | Merck Millipore | 1:400 |

**Supplementary Table 3. Possible outcomes of the αSyn-SAA based on three CSF replicates.**

| **Well Result** | | | **Qualitative Result** | **Synucleinopathy status** |
| --- | --- | --- | --- | --- |
| Type-1 | Type-1 | Type-1 | Positive | Type-1 |
| Type-1 | Type-1 | Type-2 | Positive | Undetermined |
| Type-1 | Type-2 | Negative | Inconclusive | Undetermined |
| Type-1 | Type-1 | Negative | Inconclusive | Undetermined |
| Type-1 | Negative | Negative | Negative | N.A. |
| Type-2 | Type-2 | Type-2 | Positive | Type-2 |
| Type-2 | Type-2 | Negative | Positive | Type-2 |
| Type-2 | Negative | Negative | Negative | N.A. |
| Negative | Negative | Negative | Negative | N.A. |

**Supplementary Table 4. Comparison of longitudinal performance of SAA- and SAA+ PD patients in clinical tests adjusted for age and sex.**

| Longitudinal analysis | Years | p-value_adj  (SAA-PD vs SAA+ PD) | p-value_adj  (SAA-PSP vs SAA+ PSP) |
| --- | --- | --- | --- |
| **UPDRS total** | 0 | **-** | 0.14 |
| **UPDRS H&Y score** | 0 | **0.01*** | **-** |
|  | 0.5 | **0.03*** | **-** |
|  | 1 | 0.08 | - |
|  | 3 | 0.06 | - |
|  | 5 | 0.49 | - |
| **Tremor score** | 0 | **0.008**** | **-** |
|  | 1 | **0.01*** | **-** |
|  | 3 | **0.01*** | **-** |
|  | 5 | 0.28 | - |
| **PIGD score** | 0 | **0.0002***** | 0.10 |
|  | 1 | **0.009**** | **-** |
|  | 3 | 0.13 | - |
|  | 5 | 0.86 | - |
| **Olfactory score** | 0 | **0.02*** | - |
|  | 2 | **0.006**** | - |
|  | 3 | **0.002**** | - |
|  | 5 | 0.31 | - |
| **LEDD** | 0 | 0.46 | - |
|  | 1 | 0.70 | - |
|  | 3 | 0.52 | - |
|  | 4 | 0.40 | - |
|  | 5 | 0.45 | - |
|  | 6 | 0.15 | - |
|  | 7 | 0.54 | - |
|  | 8 | 0.32 | - |
| **MMSE score** | 0 | 0.62 | - |
|  | 0.5 | **0.05*** | - |
|  | 1 | 0.96 | - |
|  | 2 | 0.29 | - |
|  | 3 | 0.94 | - |
|  | 4 | 0.85 | - |
|  | 5 | 0.64 | - |
|  | 6 | 0.97 | - |
|  | 7 | 0.28 | - |
|  | 8 | 0.42 | - |

Adjusted *p*-values were obtained through linear regression models corrected for age and sex.

**
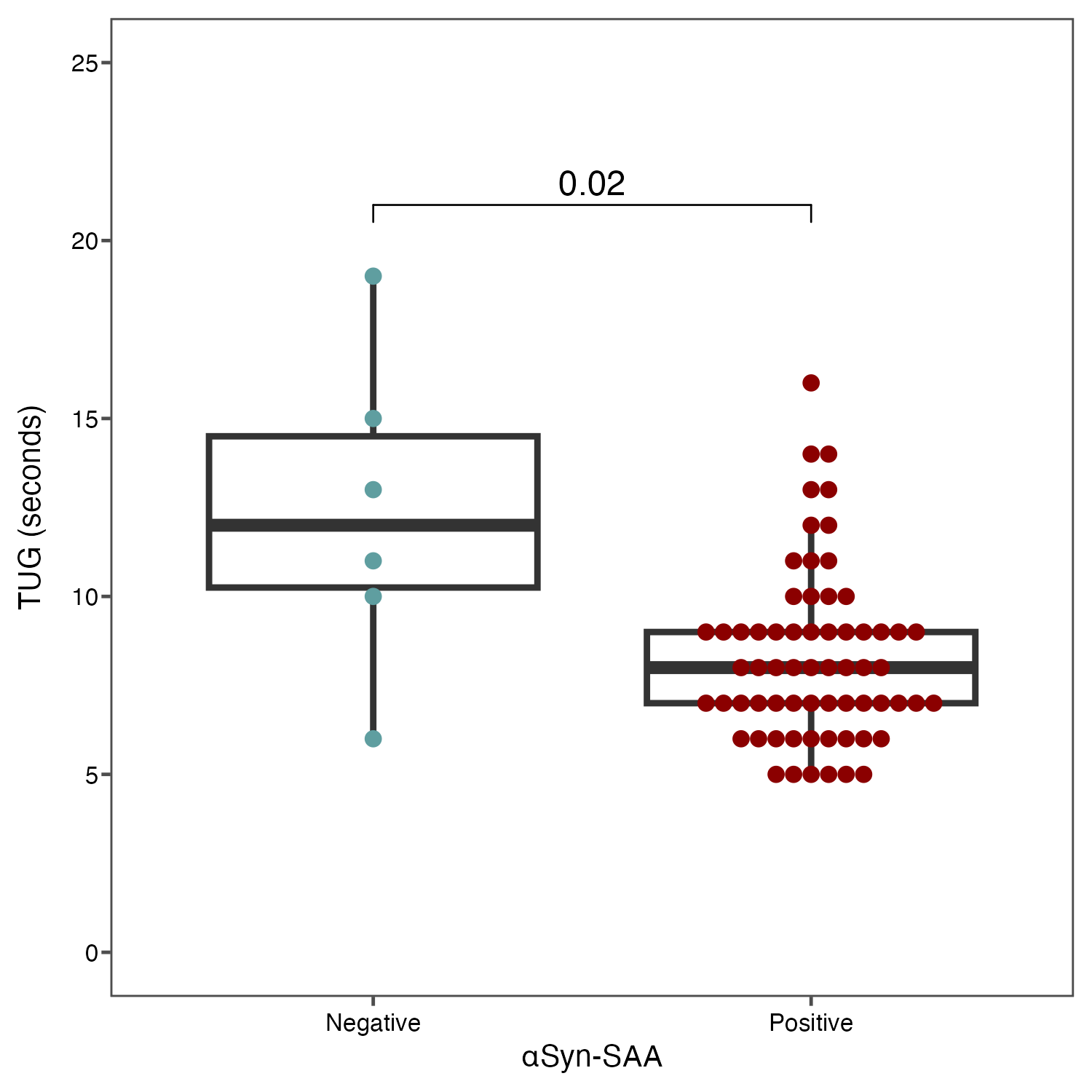
**

**Supplementary Figure 1. Time Up and Go (TUG) test in PD patients.** Comparison of the time to complete the TUG task between SAA- PD and SAA+ PD patients. Abbreviations: TUG, time up and go.

**Supplementary Table 5. Comparison between SAA- and SAA+ PD levels in DAT uptake, Aβ42 levels, and diagnostic red flags adjusted for age and sex.**

| Variable | p-value_adj  (SAA-PD vs SAA+ PD) | p-value_adj  (SAA-PSP vs SAA+ PSP) |
| --- | --- | --- |
| Most affected caudate DAT uptake | 0.10 | - |
| Most affected putamen DAT uptake | **0.0004***** | **-** |
| Amyloid-β_42_ | **0.01*** | **0.02*** |
| Neurofilament light chain | - | 0.08 |
| Early postural instability (diagnostic red flag) | **0.05*** | - |
| Symmetric parkinsonism (diagnostic red flag) | **0.01*** | - |
| Neurological status abnormalities (diagnostic red flag) | **0.02*** | - |

Adjusted *p*-values were obtained through linear regression for continuous variables and logistic regression models for binary variables corrected for age and sex.

**Supplementary Table 6. Autopsy-confirmed individuals.**

| Patient nr | SAA result | Clinical Diagnosis | Autopsy diagnosis | Pathologies found at autopsy |
| --- | --- | --- | --- | --- |
| Patient 6 | Negative | PD | AD-PSP | No LB; presence of amyloid-β deposits, p-tau and Tau-4R inclusions |
| Patient 17 | Positive | PD | PD | Typical PD pathology |
| Patient 18 | Positive | PD | PD-AD | No cortical LB; presence of AD pathology, Braak stage 3. |
| Patient 19 | Positive | PD | PD | Neocortical LB spread |
| Patient 20 | Positive | PD | PD | No cortical LB |
| Patient 21 | Positive | PD | PD | Neocortical LB spread |
| Patient 22 | Positive | PD | PD | Incipient neocortical LB spread |
| Patient 23 | Positive | PD | PD | Neocortical LB spread |
| Patient 14 | Positive | PSP | PD-AD | Neocortical LB spread; AD pathology |
| Patient 16 | Positive | PSP | PSP | Typical PSP pathology |
| Patient 24 | Negative | PSP | PSP | Typical PSP pathology |


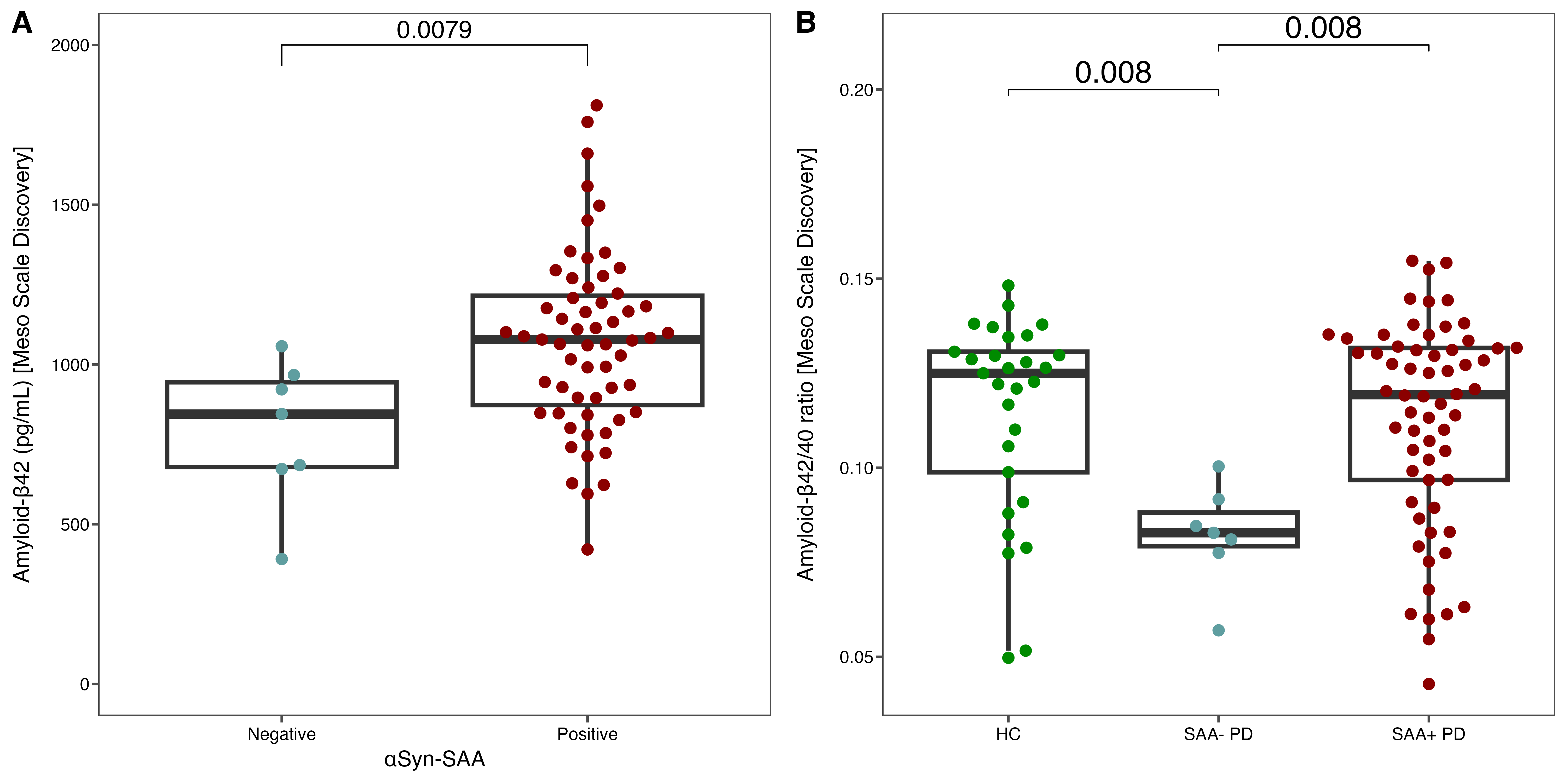


**Supplementary Figure 2. (A)** Boxplot comparing the levels of CSF Aβ_42_ between SAA- and SAA+ PD patients, as measured by a second Aβ_42_ assay (MSD® Aβ Triplex Assay). **(B)** Boxplot comparing the CSF Aβ42/40 ratio values (MSD® Aβ Triplex Assay) in SAA+ and SAA- PD patients as well as neurologically healthy individuals (HC). Adjustments for age and sex did not change the outcome of the analysis.

**Supplementary Table 7. Linear regression model for cognitive domains for SAA+ PD patients versus SAA- PD.**

|  | Estimate (SE) | Unadjusted *P*-value | Adjusted *P*-value |
| --- | --- | --- | --- |
|  | **Episodic memory** | | |
| Intercept | -1.03 (0.41) | - | **0.01*** |
| scale(Age) | -0.38 (0.12) | - | **0.002**** |
| Sex (male) | -0.64 (0.23) | - | **0.007**** |
| SAA+ | 0.62 (0.39) | **0.03*** | 0.12 |
|  | **Language** | | |
| Intercept | -0.30 (0.44) | - | 0.48 |
| scale(Age) | -0.35 (0.13) | - | **0.008**** |
| Sex (male) | -0.18 (0.24) | - | 0.46 |
| SAA+ | 0.01 (0.42) | 0.56 | 0.96 |

Adjusted *P*-value pertains to multivariate linear regression (SAA + Age + Sex) and unadjusted *P*-value was generated using univariate linear regression model for SAA outcome only.


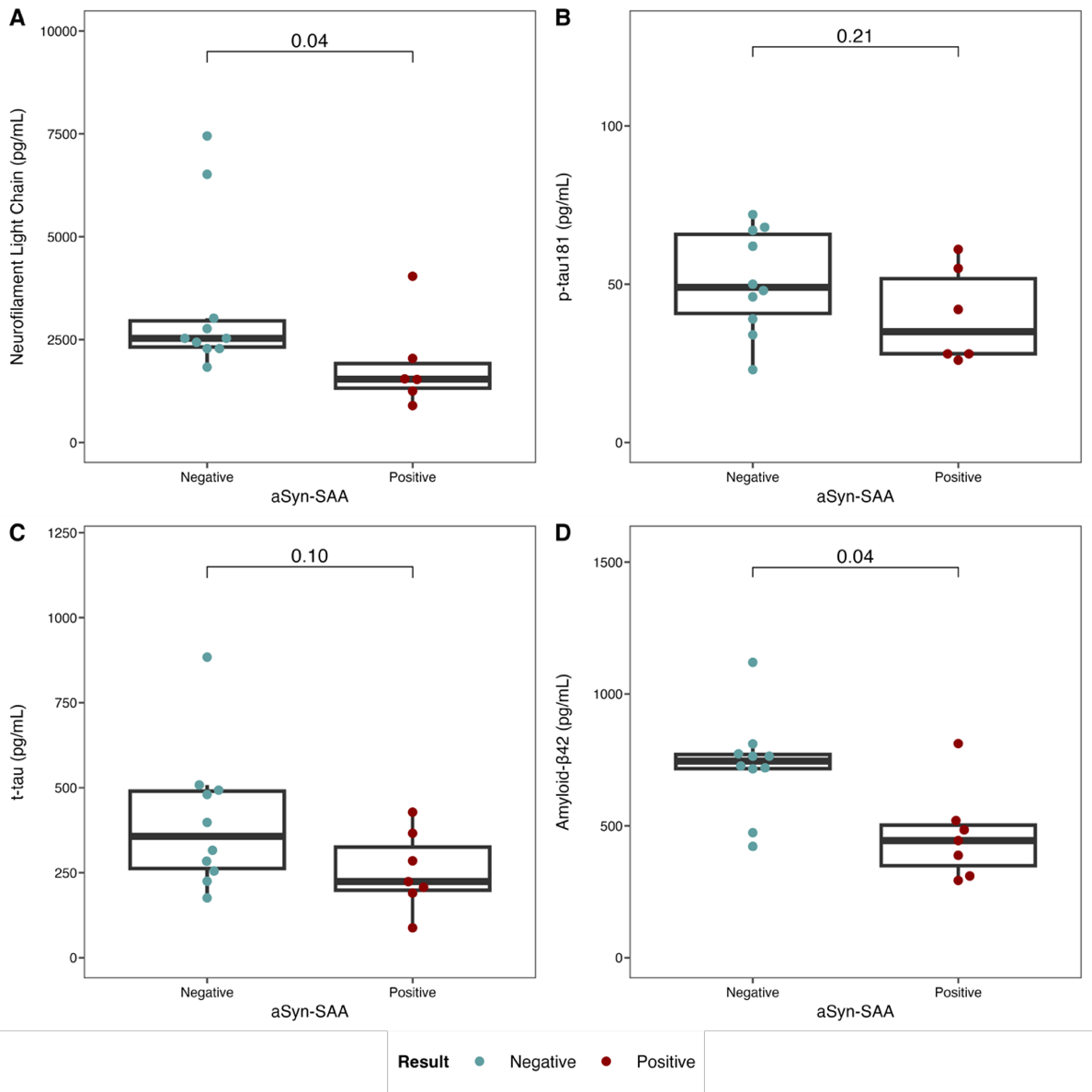


**Supplementary Figure 3. CSF AD core biomarkers in SAA+ and SAA- PSP patients. (A)** NfL; **(B)** p-tau181; **(C)** t-tau; **(D)** Aβ_42_.
